# Antioxidant Capacity in Fuchs’ Dystrophy and Its Relationship with Cataract: A Pilot Study

**DOI:** 10.3390/jcm15051773

**Published:** 2026-02-26

**Authors:** Mónica Hernández-Hernández, Mari Carmen García-Domene, Mariola Penadés, Cristina Peris-Martínez

**Affiliations:** 1 Escuela de Doctorado, Universidad Católica de Valencia San Vicente Mártir, (Doctoral School, Catholic University of Valencia San Vicente Mártir), Plaza de San Agustín, 3 Esc. A Entresuelo 1, 46002 València, Spain; moherher@mail.ucv.es; 2 Fundación de Oftalmología Médica de la Comunitat Valenciana, Avenida Pío Baroja 12, 46015 València, Spain; mariola.penades@uch.ceu.es (M.P.); cristina.p.peris@uv.es (C.P.-M.); 3 Department of Optics and Optometry and Vision Sciences, Physics School, University of Valencia, Avinguda Vicent Andrés Estellés 19, 46100 Burjassot, Spain; 4 Surgery Department, Ophthalmology, School of Medicine, University of Valencia, Av. Blasco Ibáñez 15, 46010 Valencia, Spain; 5 Biomedic Science Department, CEU Cardenal Herrera University, Calle Santiago Ramón y Cajal s/n, 46115 Alfara del Patriarca, Spain; 6 Aviñó-Peris Eye Clinic, Av. de l’Oest 34, 46001 València, Spain

**Keywords:** Fuchs’ endothelial dystrophy, cataract, antioxidant capacity, oxidative stress, corneal oedema, inflammation

## Abstract

**Background/Objectives:** The aim of this study was to compare the level of total proteins and antioxidant capacity in the corneal endothelium of patients with endothelial decompensation, due or not due to a Fuchs’ endothelial dystrophy (FECD), and analyze the influence of cataract formation. **Methods:** Endothelial cells from 27 patients with endothelial dystrophy and 6 from healthy human donors were used, distributed into four groups according to the presence of Fuchs’ Dystrophy and cataract. **Results:** Protein levels differed significantly among the five study groups (Kruskal–Wallis H = 12.19, *p* = 0.016). Patients with FECD, particularly those with concomitant cataract, showed the highest median protein concentrations, whereas lower values were observed in non-FECD groups. Post hoc Dunn’s test revealed a significant difference only between the non-FECD with cataract group and the controls (*p* < 0.05). The antioxidant capacity/protein ratio showed a marked variability across groups, with higher median values in non-FECD patients and greater dispersion in cataract groups; however, no statistically significant differences were detected (H = 5.58, *p* = 0.134). These findings highlight the differences in protein content and antioxidant defenses related to FECD and cataract status. **Conclusions:** Fuchs’ endothelial dystrophy is associated with higher protein levels and a moderately elevated but heterogeneous antioxidant capacity in the corneal endothelium, reflecting adaptive responses to chronic oxidative stress. In contrast, no FECD eyes, particularly those with cataract, showed lower or more variable antioxidant capacity, indicating reduced or inconsistent protective mechanisms.

## 1. Introduction

Corneal oedema due to endothelial decompensation is a common condition, particularly in older adults, and represents a frequent cause of visual impairment in this population. It occurs when the endothelium loses its ability to regulate hydration and maintain corneal transparency [1]. Affected patients often present with blurred vision, halos around lights, mild ocular pain, and light sensitivity, reflecting fluid accumulation in the cornea and endothelial dysfunction. This condition can develop because of Fuchs’ endothelial dystrophy (FECD), a progressive corneal disease that causes endothelial accelerated cell loss and *guttae* formation, or it may be associated with cataract, since lens opacity and related oxidative stress can exacerbate endothelial dysfunction [2]. Thus, corneal oedema serves both as a clinical manifestation of endothelial failure and as a risk factor for the progression of these pathologies, impairing vision and increasing the likelihood of surgical interventions such as corneal transplantation or cataract extraction [3,4].

Among the conditions leading to such endothelial failure, FECD is currently the leading cause of corneal transplantation in the United States and other developed countries [5]. This autosomal dominant hereditary disease primarily affects the corneal endothelium, the innermost cellular layer of the cornea responsible for maintaining its transparency by regulating hydration [6]. FECD is classified into two main forms: early-onset FECD, which manifests during the first decade of life, and late-onset FECD, the more prevalent form, which primarily affects women over 40 years of age [7]. This dystrophy is characterized by progressive dysfunction of the corneal endothelium, leading to a loss of cell density and the formation of focal excrescences on Descemet’s membrane, known as guttae, an abnormal accumulation of collagenous basement membrane material in the central cornea, resulting from stress experienced by the endothelial cells [6]. These structural alterations are responsible for the development of corneal oedema, which reduces visual acuity and, in advanced stages, can lead to blindness [8].

Oxidative stress is a critical factor in the pathogenesis of FECD. The corneal endothelium is particularly vulnerable to this type of damage due to its high metabolic activity, required to perform its ion pump and barrier functions, which demand substantial oxygen consumption. Oxidative stress manifests through the accumulation of reactive oxygen species (ROS), resulting in cellular damage and contributing to disease progression [8]. In this context, the antioxidant capacity of the corneal endothelium plays a crucial role in neutralizing the harmful effects of ROS and other free radicals. Circulating Endothelial Cells (CECs) possess several antioxidant mechanisms, including peroxiredoxins (Prx), Nicotinamide Adenine Dinucleotide Phosphate (NADPH), and NADPH quinone oxidoreductase 1 (NQO1), which help reduce ROS accumulation and protect cells from oxidative damage. However, in FECD, an oxidant–antioxidant imbalance has been observed, resulting in increased susceptibility to oxidative damage. This imbalance between oxidative and antioxidant processes contributes to DNA damage in endothelial cells, which can induce apoptosis and play a key role in disease progression [9].

In this regard, measuring total protein concentration and antioxidant capacity in corneal endothelial tissue biopsies from patients with FECD is essential to understand the pathophysiology of the disease. These measurements allow for the evaluation of oxidative stress and the antioxidant imbalance in endothelial cells, which contribute to cellular dysfunction and apoptosis. Furthermore, they help to identify molecular biomarkers associated with disease progression, facilitating early diagnosis and the assessment of targeted therapies.

On the other hand, FECD is frequently associated with a higher incidence of cataract, although a direct causal relationship between the two has not been established. This association is partly because both conditions commonly occur in elderly individuals, increasing the likelihood of their coexistence [7]. Moreover, it has been observed that both FECD and cataract share mechanisms related to oxidative stress [6], suggesting that they may be influenced by similar cellular processes. In patients with FECD, cataract surgery may accelerate corneal endothelial damage, thereby exacerbating disease progression due to inflammation, even at subclinical levels [10]. Thus, it is important to consider this association when evaluating biochemical markers related to oxidative stress. To this end, a comparative study was conducted to assess total protein levels and antioxidant capacity in corneal oedema due to endothelial decompensation, with and without FECD, also considering whether patients presented with cataract or not.

## 2. Materials and Methods

**Figure 1:**
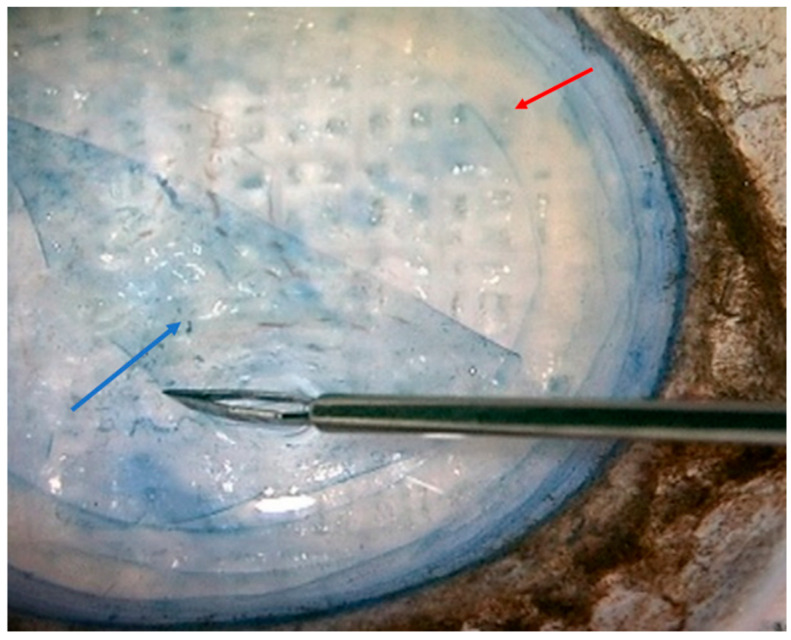
Image of a cadaveric donor cornea viewed from its concave side, where the first layer we observe is the corneal endothelium stained with 0.15% trypan blue dye (MembraneBlue™ Dutch Ophthalmic, Exeter, NH, USA). Red arrow: location of the peripheral endothelium (donut-shaped) from a cadaveric donor cornea used for DMEK corneal transplantation. The peripheral rim contains endothelial cells and is usually discarded. Blue arrow: This circular tissue is used for transplantation into the patient.

Corneal endothelial tissue samples were obtained from 27 patients with corneal oedema due to endothelial decompensation, with or without FECD and with or without cataract. Patients with other systemic or ocular pathologies that could alter the results were excluded from the study. Each sample consisted of a 9 mm circle made up exclusively of the altered endothelium and Descemet’s membrane from the central DMEK graft. Additionally, a control group consisting of six samples of cornea from a cadaveric donor was included. Each sample consists of the healthy endothelium peripheral corneal rim not used in the recipient patient for DMEK (Figure 1).

All clinical assessments, including the diagnosis of cataract and FECD, were performed by a single experienced ophthalmologist (CPM) to ensure diagnostic consistency. Cataract evaluation was conducted via slit-lamp examination using the Lens Opacities Classification System (LOCS III) utilizing retro-illumination images to identify the presence or absence of a corticonuclear component; notably, posterior subcapsular cataracts were excluded as their etiology is more closely linked to medication history than to senility. Concurrently, FECD, corneal decompensation, and oedema were diagnosed through specular microscopy and pachymetry [11].

The patient sample was distributed as follows:

G1 = FECD and cataract (n = 10), G2 = FECD without cataract (n = 4), G3 = with endothelial decompensation (no FECD) and cataract (n = 9), G4 = patients with endothelial decompensation (no FECD) without cataract (n = 4) and G5 = control group (n = 6).

All samples were obtained during Descemet membrane endothelial keratoplasty (DMEK) at Fundación de Oftalmología Médica (FOM). Table 1 summarizes the demographic and clinical characteristics of the study groups, including sample size, mean age, gender, distribution, and laterality (right or left eye). The extracted samples were stored in an Eppendorf tube at −80 °C in a liquid nitrogen container. This study adhered to the tenets of the Declaration of Helsinki for Research Involving Human Subjects Patients signed informed consent prior to their inclusion in the study.

For protein quantification, corneal endothelial samples were weighed and maintained at low temperature to prevent enzymatic degradation. The tissue was placed in a tube containing an appropriate protein extraction buffer composed of 50 mM Tris-HCl (pH 7.4), 150 mM NaCl, 1 mM EDTA, 1 mM PMSF (protease inhibitor cocktail), and 0.1% Triton X-100.

For protein quantification, corneal endothelial samples were weighed and maintained at low temperature to prevent enzymatic degradation. The tissue was placed in a tube containing an appropriate protein extraction buffer composed of 50 mM Tris-HCl (pH 7.4), 150 mM NaCl, 1 mM EDTA, 1 mM PMSF (protease inhibitor cocktail), and 0.1% Triton X-100. Homogenization was performed using a TissueLyser LT homogenizer (Qiagen, Hilden, Germany). Following cell lysis, the samples were centrifuged at 12,000× *g* for 10 min at 4 °C to obtain supernatants containing the soluble protein fraction. Total protein levels were determined using the Bicinchoninic Acid (BCA) Protein Assay Kit (Sigma-Aldrich, San Luis, MO, USA), following the manufacturer’s protocol.

Total antioxidant capacity (TAC) was measured using a commercial Antioxidant Assay Kit (Cayman Chemical, Ann Arbor, MI, USA) according to the manufacturer’s instructions. Briefly, the assay is based on the ability of antioxidants in the sample to inhibit the oxidation of ABTS to ABTS^+^ by metmyoglobin. The antioxidant capacity of the samples to prevent ABTS oxidation was compared with that of Trolox, a water-soluble vitamin E analog, and results were expressed as Trolox equivalent molar concentrations per mg of total protein. The amount of ABTS^+^ formed was quantified by measuring absorbance at 405 nm, which is directly proportional to its concentration.

A descriptive and comparative analysis of the results was performed using GraphPad Prism version 10.0.0 for Windows (GraphPad Software, Boston, MA, USA; www.graphpad.com accessed on 31 December 2025). Medians with interquartile ranges were calculated, and group comparisons were performed using the Kruskal–Wallis test, followed by Dunn’s multiple comparisons post hoc test when appropriate.

## 3. Results

### 3.1. Protein Levels According to the Presence of FECD and Cataract

**Figure 2:**
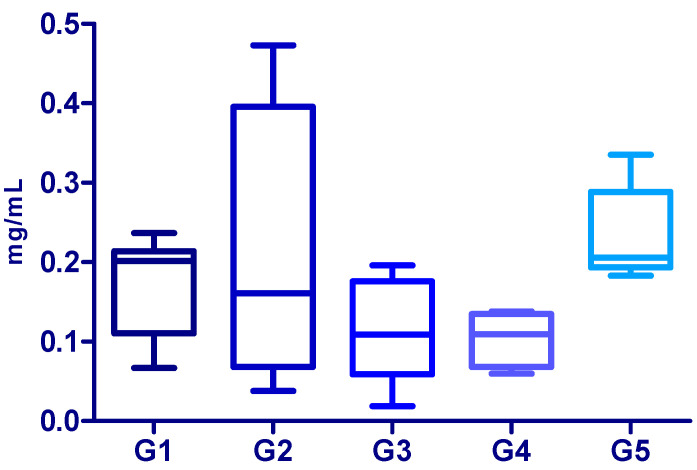
Median and interquartile range of protein levels according to the presence of FECD and cataract. G1 = FECD and cataract (n = 10), G2 = FECD without cataract (n = 4), G3 = with endothelial decompensation (no FECD) and cataract (n = 9), and G4 = patients with endothelial decompensation (no FECD) without cataract (n = 4). The control group, G5, consists of six healthy human corneas from cadaver donors.

Figure 2 illustrates the distribution of protein levels across different patient subgroups classified by the presence of Fuchs’ endothelial dystrophy and cataract. Each box represents the interquartile range (25th to 75th percentiles), the central line indicates the median, and the whiskers show the minimum and maximum values within the range, excluding outliers.

Comparing the medians, it was observed that patients with FECD with cataract exhibited the highest median value (0.2015 mg/mL) meaning this group has the highest central concentration values. Patients with FECD and without cataract and the control group show intermediate medians, relatively close to each other. These findings highlight that the presence of FECD is associated with elevated protein levels in the corneal endothelium, regardless of the coexistence of cataract (Figure 2).

In contrast, the median protein levels were lower in the no FECD groups, with values of 0.1090 in the non-Fuchs’ with cataract group and 0.1095 in the non-Fuchs’ without cataract group (Figure 2). The Kruskal–Wallis test revealed a significant difference in protein levels among the five groups (H = 12.19, *p* = 0.016). Post hoc analysis using Dunn’s multiple comparison test showed a significant difference only between group G3 and group G5 (*p* < 0.05), with the median protein level of the group without FECD (G3) being significantly lower than that of the control group (G5). No other pairwise comparisons reached statistical significance. These results suggest that, although there may be biologically relevant variation in protein levels among the different clinical groups, this variation was not consistent or pronounced enough to be detected across most comparisons, likely due to the small sample size in each group.

### 3.2. Total Antioxidant Capacity/Protein Ratio

The ratio between total antioxidant capacity and protein concentration in corneal endothelial samples was compared across five groups: patients with Fuchs’ endothelial dystrophy with and without cataract as well as patients without dystrophy with and without cataract (Table 2).

The group of patients with FECD and cataract (n = 10) showed a median value of 6.77 with a wide range of 15.9. Values ranged from 5.94 to 21.8, displaying a dispersed distribution pattern that reflects heterogeneity in the evaluated ratio. In patients with FECD but without cataract (n = 4), the median was 8.10, also with a large dispersion (range of 32.4). This group exhibited the highest maximum value among the Fuchs’ subgroups (35.1), which may have been influenced by the small sample size.

The group of patients without FECD but with cataract (n = 11) had a median of 11.6. This group also showed the widest range among all groups (67.5), with extreme values reaching up to 72.3. The high variability is mainly due to very small sample sizes (Table 2).

On the other hand, the group without FECD or cataract (n = 4) presented a median of 12.0, with a lower dispersion (range of 12.5) compared to the previous group. The control group exhibited the lowest values. These results suggest a more stable profile in terms of antioxidant balance in individuals without FECD or cataract (Table 2). However, the Kruskal–Wallis test yielded a value of 5.577; df = 3; *p* = 0.1341, indicating no statistically significant differences in antioxidant capacity among the groups.

## 4. Discussion

Understanding the biochemical alterations associated with FECD is essential for elucidating the mechanisms underlying endothelial dysfunction and for guiding early diagnosis and targeted therapeutic strategies. The present study provides insights into protein expression and antioxidant capacity in the corneal endothelium, highlighting potential cellular responses to oxidative stress and their variability among patients with and without cataract. The inclusion of a control group in this study allowed for the establishment of baseline levels of protein and antioxidant capacity in healthy corneal endothelium. These reference values provided a physiological benchmark against which the biochemical alterations observed in FECD and cataract groups could be compared. Given the small and uneven sample sizes, particularly in some subgroups, this study should be considered exploratory and hypothesis-generating. The findings provide descriptive insights but cannot support definitive conclusions regarding protein expression or antioxidant capacity in FECD or cataract.

The results of this study show that protein concentrations in the corneal endothelium vary according to the presence of Fuchs’ endothelial dystrophy and cataract. Notably, protein levels in FECD patients showed a descriptive trend toward higher values compared to controls, although these differences did not reach statistical significance. This increase in protein content may be associated with endothelial decompensation, oxidative stress, or tissue repair responses characteristic of the disease, reflecting abnormal activation of cellular pathways triggered by chronic endothelial stress [12,13]. When compared to the control group, protein concentrations in the non-Fuchs’ sub-groups were significantly lower, whereas those in the FECD groups showed a descriptive trend toward higher levels compared to controls, without reaching statistical significance. This observation indicates a descriptive trend toward increased protein expression in FECD, but only the difference between non-FECD cataract and control groups reached statistical significance.

In contrast, the lowest protein levels were observed in the group without FECD but with cataract. It is generally known that corneal endothelial protein levels tend to be lower in elderly patients [14,15]. This situation may impact the levels of ocular proteins necessary for proper eye function.

Nevertheless, we acknowledge that total protein measurements may be affected by factors such as variability in tissue size, endothelial cell density, extent of Descemet membrane removal, and potential stromal contamination. While care was taken to standardize tissue processing, future studies incorporating normalization to endothelial cell density, quantitative proteomic approaches, or histological verification of tissue composition would be valuable to further substantiate these findings and refine the biological interpretation of protein alterations in FECD and cataract.

The total antioxidant capacity/protein ratio was used as an exploratory parameter to compare relative antioxidant status across the different clinical conditions evaluated. While this approach allows partial normalization for variations in protein content, it remains sensitive to several technical and biological factors, including differences in sample volume, endothelial cell number, tissue composition, and potential contamination by stromal or inflammatory cells. Therefore, the findings should be interpreted cautiously and primarily as descriptive indicators of relative antioxidant balance rather than as direct measures of antioxidant efficiency.

Within these constraints, the results indicate a descriptive trend toward a moderate but heterogeneous antioxidant profile in FECD, which should be interpreted cautiously given the limited sample size and high inter-individual variability. The wide variability observed among FECD patients may reflect inter-individual differences related to disease stage, comorbidities, or genetic background, and is consistent with the concept of a chronically stressed endothelium exhibiting variable adaptive responses to oxidative challenge [15,16]. However, the present data do not allow direct mechanistic conclusions regarding the activation or effectiveness of specific antioxidant pathways.

In patients without FECD, total antioxidant capacity also showed marked variability, particularly in those with cataract. This finding may be consistent with inter-individual differences in endogenous antioxidant responses rather than a uniform disease-related effect. In contrast, individuals without FECD or cataract exhibited a lower variability in the TAC/protein ratio, suggesting a more stable antioxidant balance under physiological conditions. However, the small and unevenly distributed groups increase the risk of type II error, meaning that true differences between groups may not have been detected. High inter-individual variability, differences in tissue composition, endothelial cell density, and potential technical variability further limit the robustness of comparisons. Therefore, the observed trends should not be interpreted as biologically definitive and should primarily serve as hypothesis-generating observations for future studies.

Previous studies have demonstrated that the human lens possesses multiple endogenous antioxidant mechanisms that help limit reactive oxygen species accumulation and delay cataract development, particularly in early stages [16]. These include intercellular communication via gap junctions composed of connexins (Cx43, Cx46, and Cx50), which facilitate the distribution of small antioxidant molecules and support lens homeostasis [17,18]. In addition, antioxidant defenses in the lens are regulated by transcriptional pathways such as Nrf2 signaling, which coordinates the expression of antioxidant enzymes and protein quality control systems [18]. While these mechanisms provide a relevant biological context, their direct involvement cannot be inferred from the assays used in this study.

Overall, the present findings indicate that antioxidant capacity in corneal endothelial samples varies widely across clinical conditions, particularly in FECD and cataract. This variability likely reflects a combination of biological heterogeneity and methodological factors. A limitation of this study is the relatively small and uneven sample size across groups, which may reduce statistical power and limit the robustness of the comparisons. Consequently, the results should be viewed as hypothesis-generating and highlight the need for future studies incorporating more specific oxidative stress markers, normalization to endothelial cell density, and targeted molecular analyses to better define the role of antioxidant responses in corneal and lens pathology.

## Figures and Tables

**Table 1:** Demographic characteristics of the sample. The sample size for each group (N), age, gender ratio, and right-to-left eye ratio are presented. G1 = FECD and cataract, G2 = FECD without cataract, G3 = with endothelial decompensation (no FECD) and cataract, and G4 = patients with endothelial decompensation (no FECD) without cataract. The control group, G5, consists of healthy human corneas from cadaver donors.

	G1	G2	G3	G4	Control Group (G5)
N	10	4	9	4	6
Age (years, Media ± SD)	73 ± 8	64 ± 9	64 ± 13	65 ± 10	64 ± 20
Masc/Fem	3/7	1/3	2/7	1/3	4/2
Right eye/Left Eye	7/3	4/1	5/4	2/2	3/3

**Table 2:** Comparison of total antioxidant capacity/protein ratio values according to the presence of FECD and/or cataract. The sample for each group (N), the median, 25th and 75th percentiles, and maximum and minimum values are presented. G1 = FECD and cataract, G2 = FECD without cataract, G3 = with endothelial decompensation (no FECD) and cataract, and G4 = patients with endothelial decompensation (no FECD) without cataract. The control group, G5, consists of healthy human corneas from cadaver donors.

	G1	G2	G3	G4	Control Group (G5)
N	10	4	9	4	6
Minimum	5.94	2.79	4.80	10.0	3.77
25th Percentile	6.35	4.06	7.27	10.1	4.61
Median	6.77	8.10	11.6	12.0	6.30
75th Percentile	13.1	28.4	21.8	20.3	6.57
Maximum	21.8	35.1	72.3	22.5	6.88

## Data Availability

The original contributions presented in this study are included in the article. Further inquiries can be directed to the corresponding author.

## Abbreviations

ABTS	2,2′-Azino-bis(3-ethylbenzothiazoline-6-sulfonic acid)
ARE	Antioxidant Response Element
BCA	Bicinchoninic Acid
CECs	Circulating Endothelial Cells
Cx43	Connexin 43
Cx46	Connexin 46
Cx50	Connexin 50
DMEK	Descemet Membrane Endothelial Keratoplasty
EDTA	Ethylenediaminetetraacetic Acid
ER	Endoplasmic Reticulum
FECD	Fuchs’ Endothelial Corneal Dystrophy
HSPs	Heat Shock Proteins
NQO1	NADPH Quinone Oxidoreductase 1
NADPH	Nicotinamide Adenine Dinucleotide Phosphate
NP-40	Nonidet P-40 (Nonionic Detergent)
PMSF	Phenylmethylsulfonyl Fluoride
ROS	Reactive Oxygen Species
SD	Standard Deviation
TAC	Total Antioxidant Capacity
Tris-HCl	Tris(hydroxymethyl)aminomethane Hydrochloride
